# How does loneliness affect satisfaction with life? What is the role of the perception of God in this interaction?

**DOI:** 10.3389/fpsyg.2025.1550108

**Published:** 2025-02-17

**Authors:** Feridun Kaya, Gülin Yazıcı Çelebi

**Affiliations:** ^1^Department of Psychology, Faculty of Letters, Atatürk University, Erzurum, Türkiye; ^2^Department of Psychology, Faculty of Letters, Gümüşhane University, Gümüşhane, Türkiye

**Keywords:** loneliness, satisfaction with life, perception of God, moderation, Turkey

## Abstract

**Introduction:**

This study examined the role of loneliness and the perception of God in affecting the satisfaction with life of Muslim individuals living alone in Turkey during the COVID-19 pandemic. Additionally, the study explored the regulatory role of the perception of God in the relationship between individuals’ loneliness and satisfaction with life.

**Methods:**

The research is a cross-sectional study that evaluates individuals’ loneliness, satisfaction with life, and perception of God. The study group consists of 378 individuals living alone in Turkey. Among the participants, 196 are women (51.9%) and 182 are men (48.1%). The UCLA loneliness scale, the satisfaction with life scale, the perception of God scale, and a personal information form were used as data collection tools in the study.

**Results:**

The examination of research findings indicated that the variables of loneliness, perception of God, and the interaction between loneliness and the perception of God explained 28% of the variance in individuals’ satisfaction with life. We determined that satisfaction with life was affected significantly and positively by the perception of God (*β* = 0.28, *p* < 0.001) and significantly and negatively by loneliness (*β* = −0.38, *p* < 0.001). The interactional effect of the variables of loneliness and perception of God on satisfaction with life was also found to be significant (*β* = −0.10, *p* = 0.023). When we examined the details of the regulatory effect, we found that the effect of loneliness on satisfaction with life decreased even more in cases where the perception of God was high.

**Discussion:**

The research findings suggest that loneliness decreases life satisfaction, while positive self-image mitigates this effect. It can be stated that using belief-sensitive therapeutic approaches in the therapeutic process could contribute to alleviating the negative effects of loneliness.

## 1 Introduction

The world met COVID-19 in late 2019. Although first impressions were very frightening, many of us could not foresee that our habits in our daily life would change in the coming days. Many routines in our lives began to change with the appearance of the virus. This change process was mainly affected by the fact that the spread of the COVID-19 virus could not be stopped and that the virus brought many variants with it. From December 2019, when the first virus case was reported, to June 2020, more than 6.7 million people were affected by the virus and more than 390,000 people were reported to have died from the virus (World Health Organization, 2020a, 2020b).

The first news of death from COVID-19 in the country came on March 15, 2020. In this process, various pharmacological treatments were tried and vaccine studies accelerated. During this period, countries implemented various measures, such as working from home, local or general quarantine, travel restrictions, and closure of schools, to control the spread of the virus (Aydin and Kaya, 2021). Researchers emphasized that governments needed to combine multiple measures and implement them on time and over a long period to prevent the spread (Kelso et al., 2009). In other words, the wrong application of the measures by countries might worsen the pandemic. Research on the subject indicated that social distancing, in particular, could help alleviate the burden of the pandemic (Kelso et al., 2009; Liu et al., 2016). Accordingly, the World Health Organization (2020c) listed a series of recommendations on personal preventive measures to reduce the spread of COVID-19. Due to the COVID-19, which affected the entire world, many countries gradually closed their borders to other countries. Also, quarantines were put into practice. Partial and full closure periods were put into practice due to the increasing number of cases and deaths during this period. There is a substantial body of research evidence indicating a relationship between loneliness and satisfaction with life during the COVID-19 pandemic (Deutrom et al., 2021; Kumar et al., 2021; Lorber et al., 2023; Onal et al., 2022).

Individuals’ satisfaction with life, which includes self-evaluation about their lives after changing periodic conditions, may also be affected for various reasons. The satisfaction with life examined in this study corresponds to a cognitive process that includes individuals’ evaluation of their life as a result of comparing their conditions and standards (Diener et al., 1985). On the other hand, loneliness, whose predictive effect on satisfaction with life will be examined in the study, can be considered as a concept that is formed as a result of individuals’ perception and evaluation of themselves and their social relations and whose cognitive side predominates. Peplau and Perlman (1982), who made significant contributions to the literature on loneliness, also discussed loneliness from a cognitive perspective. Young (1982), on the other hand, emphasized that loneliness was related to irrational beliefs and stated that erroneous beliefs would increase loneliness. Similarly, loneliness is seen as a result of perceived social isolation and this situation leads to excessive alertness towards (additional) social threats in the environment (Cacioppo et al., 2006). In other words, unconscious surveillance of a social threat brings with it cognitive biases. For example, lonely individuals see the social world as a more threatening place than individuals who are not lonely, expect more negative social interactions, and remember more negative social information (Cacioppo and Hawkley, 2009; Hawkley and Cacioppo, 2010). On the other hand, the perception of God, whose role as a moderator was examined in the study, is a concept with a cognitive dimension that includes the attributions and thoughts of the individual about God. Guthrie (2001) stated that there were many views that religion itself was generally a kind of cognition. In addition, according to researchers, holding beliefs strongly, whether referring to the existence or non-existence of God, can have a beneficial effect on its own by reducing cognitive contradiction and positively affect an individual’s well-being (Villani et al., 2019). The concepts of both satisfactions with life, loneliness, and the perception of God examined in the research are concepts that are shaped as a result of individuals’ perceptions and interpretations and have strong cognitive aspects. For this reason, this study was based on the cognitive approach. The basic idea of the ABC model, which is handled within the framework of the cognitive approach, shows that external events (A) cannot cause emotions (C), but beliefs can (B) (DiGiuseppe et al., 2014). In this context, first, we think that negative beliefs and thoughts (B) towards loneliness (A) may cause individuals to perceive their satisfaction with life (C) negatively. Secondly, we can say that the positive perceptions and thoughts of individuals (B) towards God (A) will contribute to the positive perception of their satisfaction with life (C). Finally, we think that in the case of loneliness (A), thoughts about the perception of God (B) will affect the satisfaction with life of individuals (C).

### 1.1 Satisfaction with life

Satisfaction with life is a multidimensional concept because it includes both cognitive and emotional evaluations of life in general (Diener and Diener, 1995; Sam, 2001). These evaluations may include issues related to business life, as well as those related to private life and close relationships. This situation also diversifies the factors that can affect satisfaction with life. Some studies have shown that factors, such as having a meaningful life, enjoying life, and having a lot of engagement, are associated with satisfaction with life (Peterson et al., 2005). In addition to these, satisfaction with life is also affected by factors, such as demographic variables, personality traits, cognitive characteristics, health status (Chow, 2005), external factors, subjective evaluations, and emotional status (Diener, 1984). Research has shown that demographic variables, such as gender, age, and perceived economic status, also affect satisfaction with life (Peterson et al., 2005; Sam, 2001). Considering that personality traits can have an impact on worldview and coping, we can say that they are also related to satisfaction with life. Some studies confirm this view (Gish et al., 2022; Udayar et al., 2020; Weber and Huebner, 2015). Similarly, cognitive processes, which are the source of emotions and behaviors, have an effect on satisfaction with life, which is closely related to the individual’s evaluations of life (Ash and Huebner, 2001; Cummins and Nistico, 2002; Mehlsen et al., 2005). In addition, relationships with others are also among factors affecting satisfaction with life (Adams et al., 1996).

### 1.2 Loneliness

Man is a social being, and one of his/her basic needs is to establish meaningful and close relationships with other people. Inability to meet this need adequately results in loneliness. According to Peplau and Perlman (1982), loneliness is the difference between individuals’ existing social relationships and the social relationships they want to have. Peplau and Perlman (1982) proposed that there are three fundamental commonalities in the definition of loneliness: First, loneliness results from an individual’s limited social relationships. Second, loneliness is a subjective experience and varies based on the individual’s perception. It can emerge when one is alone or even in a crowd. Third, the experience of loneliness is unpleasant and distressing. Weiss (1973) examined loneliness under two categories: emotional and social loneliness. Social loneliness refers to the perceived inadequacy in social relationships and the individual’s failure to feel like a part of the group, event, or activity when sharing activities with others in their environment. Emotional loneliness, on the other hand, is when an individual does not perceive themselves as emotionally close to another person, feels distant, and senses a lack of acceptance (Diehl et al., 2018).

In the literature, loneliness is discussed in three categories, namely short-term, situational, and chronic loneliness (Saporta et al., 2021). Situational and short-term loneliness can be considered normal, while chronic loneliness can have negative effects on the individual, cause cognitive and emotional difficulties, and have consequences leading to pain and hopelessness. During the COVID-19 pandemic, quarantine measures were implemented worldwide to prevent the spread of the disease, and during this period, people maintained a social life limited to their household. Individuals living alone, however, remained entirely on their own. It is believed that the social isolation initiated by this quarantine process could be a more challenging experience for individuals living alone. Overall, COVID-19 is reported to have had negative effects on mental health (Nilima et al., 2021; Schafer et al., 2022; Xiong et al., 2020). Schluter et al. (2022), in their cross-sectional study conducted with participants from eight countries, concluded that quarantine and/or isolation measures during the pandemic were associated with significant mental health deterioration. Researchers have reported that the pandemic directly affected psychopathology regardless of gender, group, or region (Cénat et al., 2021; Demartini et al., 2020; Schafer et al., 2022).

Jaramillo and Felix (2023), using a qualitative constructivist theoretical approach to identify the psychosocial effects of the COVID-19 pandemic and develop empirical insights, identified five themes in their study. These themes revolved around mental health experiences, family factors, pandemic-related communication, career/academic disruptions, and systemic/environmental factors. It is evident that the circumstances brought about by the pandemic have affected multiple areas of life. Studies reveal that social distancing and lockdowns implemented during the pandemic led to increased reports of loneliness (Chiesa et al., 2021; Hoffart et al., 2020; Ivbijaro et al., 2020) and that social isolation is associated with loneliness and satisfaction with life (Clair et al., 2021). The COVID-19 pandemic and its aftermath have brought existential issues, often overlooked or avoided, to the surface. Research indicates that quarantine measures have caused psychological harm and triggered emotions such as loneliness (Brooks et al., 2020; Dagnino et al., 2020; Ganesan et al., 2021). In general, loneliness is reported to pose a risk, particularly for older adults (Donovan and Blazer, 2020; Fakoya et al., 2020). Based on the idea that loneliness may affect satisfaction with life, loneliness has been included as a variable in this research.

### 1.3 Relationships between loneliness and satisfaction with life

There are many research findings in the literature that reveal a significant relationship between loneliness and satisfaction with life (Andrew and Meeks, 2018; Moore and Schultz, 1983; Sezer and Oktan, 2020; Schumaker et al., 1993; Swami et al., 2007; Tuzgöl Dost, 2007; Yıldırım-Kurtuluş et al., 2023). The majority of studies show a significant negative relationship between satisfaction with life and loneliness although they have been conducted with different age groups, in different cultures, and under different conditions (Ozben, 2013; Kearns et al., 2015; Hu et al., 2016). Similar to this study, a study conducted with 466 participants in Switzerland during the COVID-19 pandemic indicated that as loneliness and well-being were negatively correlated and that the level of well-being decreased as the level of loneliness increased (Gubler et al., 2021). In the literature, the concepts of satisfaction with life, happiness, and well-being are frequently used interchangeably. According to Diener (2009), satisfaction with life is the most important indicator of subjective well-being. In addition, many studies conducted before the pandemic revealed that there was a significant inverse correlation between loneliness and satisfaction with life. Some studies, including the study conducted by Cava et al. (2018) with adolescents aged between 12 and 17, Guignard et al. (2021) with gifted adolescents, Gan et al. (2020) with university students, Yukay-Yüksel et al. (2020) with young adults, Fernández-Alonso et al. (2012) with adult women, and Bai et al. (2018) with elderly individuals, showed that satisfaction with life decreased with the increase in loneliness. In addition to studies in which cross-sectional data had been used, the review of studies using longitudinal data indicated that satisfaction with life decreased with the increase in loneliness (Marttila et al., 2021; Nie et al., 2020; Tough et al., 2018). All these study results reveal that loneliness can be a significant predictor of satisfaction with life.

### 1.4 Regulatory and moderator role of the perception of god

One of the factors that will be effective in combating adverse situations in an individual’s life is the religious beliefs and the perception of God. For example, Calvert (2010) concluded that having a close and secure relationship with God was positively related to psychological health and that this relationship contributed to the well-being of individuals. Also, Kelly (2020) concluded that devotion to God had a healing effect on loneliness. In the literature, research findings indicate significant relationships between the perception of God and loneliness (Le Roux, 1998; Scott et al., 2014). The perception of God is defined as the image of God in the mind of the individual, which includes all the emotions, thoughts, and references to God (Güler, 2007). Lawrence (1997) defined the perception of God as the chain of meanings attributed to the concept of God by the individual. Perceptions of God can be positive or negative; God can be perceived as forgiving and merciful by some and as punishing and unforgiving by others (Güler, 2007).

In the literature, many studies have investigated the effects of relationships with God, spirituality, secure attachment to God, perception of God, and similar variables on the individual (Bonhag and Upenieks, 2021; Bradshaw et al., 2010; Leman et al., 2018). In addition, findings suggest that a negative perception of God is associated with higher levels of distress and greater depression (Schaap-Jonker et al., 2017). Stulp et al. (2019), in a meta-analysis study conducted using a dataset of 29,963 individuals, concluded that a positive perception of God is associated with well-being, while a negative perception of God is associated with psychological distress. It appears that the perception of God is related to various other variables. For example, Le Roux (1998) concluded that individuals who had a strong faith experienced less loneliness and stated that loneliness mainly occurred when individuals broke their connections with God. Similarly, Kelly (2020) concluded that devotion to God would have healing effects on loneliness. Studying similar variables, Cosan (2014) concluded that there was a negative relationship between loneliness and satisfaction with life. Numerous research findings indicate that the concepts of spirituality and religiosity significantly predict satisfaction with life (Esteban et al., 2021; Jafari et al., 2010; Marques et al., 2013).

Kirkpatrick and Shaver (1992) concluded that individuals who defined their relationship with God as a ‘secure attachment’ had higher levels of satisfaction with life and lower levels of loneliness. In addition, studies on the relationship between religion and spirituality and loneliness have shown that loneliness and spirituality are inversely correlated (Koenig et al., 2012). In addition, many studies have shown that there is a relationship between the perception of God and satisfaction with life (Benson and Spilka, 1973; Plouffe and Tremblay, 2017; Szcześniak et al., 2019; Zarzycka and Zietek, 2019). These results indicate significant relationships between variables. Piety, which can be defined as having strong ties with God and loyalty to the provisions of the religion to which one belongs, is evaluated as a concept associated with a positive perception of God (Kalmykova, 2021). In other words, religious individuals’ positive perception of God is significantly higher than the perception of those who are not religious. In the literature, some research results show that religiosity is positively correlated with satisfaction with life (Cohen et al., 2005; Lim and Putnam, 2010) and negatively with loneliness (Baumeister and Storch, 2004; Kirkpatrick et al., 1999). In their study on the relationship between the image of God and loneliness, Schwab and Petersen (1990) concluded that there was a positive correlation between loneliness and the image of an angry God, and a negative correlation with the image of a helpful/compassionate God. The result of the longitudinal research in which religion and loneliness were addressed revealed that the religious involvement of individuals reduced their loneliness (Upenieks, 2022). In addition to the quantitative studies, qualitative research results, which provide an in-depth examination of the research subject, have shown that religiosity protects individuals from loneliness (Ciobanu and Fokkema, 2017). This situation raises the question of whether the moderator role of the perception of God is significant in the relationship between loneliness and satisfaction with life.

The perception of God, which was examined as the regulatory/moderator variable of the research, is associated with the schemas related to God in the mind of the individual (Güler, 2007). According to Seyhan (2014), the perception of God is largely related to what a person thinks and has learned about God, and from this point of view, it is a concept that is shaped at the cognitive level. From the point of view of the cognitive approach on which this research was based, schemas are shaped by the information acquired from childhood and can shape perceptions (Young et al., 2003). These schemas are influenced by the culture in which one lives, past experiences, and environmental factors (Boutyline and Soter, 2021). Perceptions of God can also be considered a type of schema. If an individual’s perception of God is forgiving, protective, and loving, this perception may serve a protective function during negative life events (McIntosh, 1995). In the context of this study, loneliness is a negative predictor of satisfaction with life. However, if the mediating variable, the perception of God, is positive, even if the individual feels lonely, they may find support in the presence of God, and the negative impact of loneliness on satisfaction with life may be mitigated. Using the ABC model as an example: A: The individual feels lonely. B: “God is everywhere, protecting and loving me.” C: When we consider satisfaction with life, the positive effect of the perception of God becomes more evident.

When God is considered a personal attachment figure, forming a positive relationship with Him can weaken the negative effects of loneliness (Minner, 2009). This relationship can function as a form of social support (Counted, 2016). Relationships with God, which are also thought to be effective in giving life meaning and helping individuals set goals, are likely to have positive effects on individuals (Jordan et al., 2021). The sample of this study consists of Muslim individuals, and it can be suggested that strong values in Islam, such as gratitude, patience, and contentment, positively influence satisfaction with life. The combination of a positive perception of God with these values is expected to alleviate the negative effects of loneliness and have a positive relationship with satisfaction with life. All religions promote solidarity, mutual assistance, and sharing among people. In this context, the relationship between loneliness and religion/faith can be exemplified.

We can say that a similar process is effective in the perception of God. From another point of view, the individual’s perception of God can also affect his/her perception and interpretation of his/her life and experiences (Koenig, 2012). We can say that an individual who perceives God as a forgiving, loving, and protective power will feel less alone and will always see God as supportive. From the point of view of the moderator effect of the perception of God, we can consider that those who have a positive perception of God, that is, those who perceive God as a forgiving, protective, and helping power, will feel God by their side in difficult times, which will reduce the feeling of loneliness and thus have positive effects on satisfaction with life (Stulp et al., 2019). We can say that those who constantly stay in contact with God through prayers or similar ways and feel God’s support will feel less lonely. This situation is thought to indirectly alleviate the detrimental effect of loneliness on satisfaction with life. In addition, studies on the subject in the literature mainly focus on Christianity and its sects, therefore, studies with individuals of different religions will contribute to the literature. Moreover, we think that examination of the role of the perception of God in reducing the effect of loneliness seen in individuals living alone during the COVID-19 process on their satisfaction with life makes this study original.

In the literature, studies have shown different kinds of relationships between loneliness, satisfaction with life, and the perception of God. We understand that the COVID-19 process has left deep traces in the lives of individuals and has an impact on their moods. In this process, research has shown that elderly individuals have difficulty overcoming COVID-19 more and that death rates of elderly individuals are higher (Antonelli et al., 2021). Considering all these, we predict that the feelings of loneliness of middle-aged and older individuals will increase during the closure period and as a result, their satisfaction with life will be affected. In addition, the negative experiences in this process, which also affect individuals’ perception of God, suggest that these individuals may experience their satisfaction with life in different ways.

In the literature, research is often conducted on the general population. However, studies focusing on samples consisting of middle-aged and older adults living alone are limited. From this perspective, it is believed that this research will contribute to the literature and fill a gap. Additionally, this study is one of the pioneering works demonstrating how the perception of God moderates the relationship between loneliness and satisfaction with life in middle-aged and older individuals. This study was conducted to determine the relationship between the loneliness levels of individuals and their satisfaction with life and the regulating role of perception of God in this relationship. For this purpose, the following hypotheses were tested in the study:

1. The predictive effect of loneliness on satisfaction with life is significant.
2. The predictive effect of the perception of God on satisfaction with life is significant.
3. The predictive effect of the interaction between loneliness and perception of God on satisfaction with life is significant.

## 2 Method

This research is a cross-sectional study that evaluates individuals’ loneliness, satisfaction with life, and perception of God. The study was conducted on individuals aged 40 and above who live alone in the provinces of Gümüşhane, Bayburt, and Erzurum. The sample was selected using simple random sampling methods (Fraenkel et al., 2012). The sample size was determined through power analysis. Using G*Power 3.1 software (Faul et al., 2009), the required sample size for multiple regression analysis was calculated as at least 107 participants, based on two predictor variables, an alpha level of 0.05, a power of 0.95, and a medium effect size (Balkin and Sheperis, 2011).

### 2.1 Study group

The study group consisted of 378 individuals, including 196 females (51.9%) and 182 males (48.1%). The ages of the participants ranged between 40 and 65 (*Mean* = 52.81, *SD* = 6.08). Table 1 shows detailed information about the participants of the study.

**Table 1:** Demographic variables of participants.

Variable		*N*	%
Gender	Female	196	51.9
	Male	182	48.1
Place of residence	City	346	91.5
	Town	22	5.8
	Village	10	2.6
Faith in God	Yes	369	97.6
	Not stated	9	2.4
Religion	Muslim	374	98.9
	Not stated	4	1.1
Level of education	Elementary school	217	57.4
	Middle school	126	33.3
	High school	35	9.03
Socio-economic level	Low	172	45.5
	Middle	146	38.6
	High	60	15.9
Chronic diseases	Yes	70	18.5
	No	308	81.5
Someone with a diagnosis of COVID-19 in the close circle	Yes	293	77.5
	No	85	22.5
Death of someone from COVID-19 in the close circle	Yes	28	7.4

### 2.2 Procedures and ethical approval

First, the ethics committee approval was obtained in the study. Ethics committee approval (E-95674917-108.99-71601) was obtained from Gumushane University. An online questionnaire was created, explaining the study’s purpose, tools, and completion time, with an informed consent section for voluntary participants. Data were analyzed using SPSS 21 and IBM AMOS 23.

### 2.3 Data collection tools

In this study, data collection tools included the UCLA Loneliness Scale, the Satisfaction with Life Scale, the Perception of God Scale, and a Personal Information Form. These tools are described below.

#### 2.3.1 The personal information form

This form was prepared by the researcher. It consists of questions about the gender, age, economic status, and religious beliefs of the participants.

#### 2.3.2 The UCLA loneliness scale

The UCLA loneliness scale, which was developed by Russell et al. (1978), was used to determine the loneliness levels of individuals in the study. The validity and reliability studies were conducted by Demir (1989) by adapting it to Turkish culture. It consists of 20 four-point Likert-type items. The scores that can be obtained from the scale range between 20 and 80, and high scoresshow intense feelings of loneliness. In the adaptation study of the scale to Turkish culture, the Cronbach’s alpha value calculated for reliability was found to be 0.96 (Demir, 1989). When examining studies conducted with the measurement tool, it was determined that the scale also produced highly reliable results in other studies. For example, in the study by Yıldırım et al. (2024), the Cronbach’s alpha value was 0.86, and in the study by Karaman and Haktanir (2024), the Cronbach’s alpha value was 0.83. For the reliability of the data obtained in this study, the Cronbach’s alpha value was calculated as 0.87, indicating that the results are highly reliable.

#### 2.3.3 The satisfaction with life scale

The Satisfaction with Life Scale was developed by Diener et al. (1985) to determine the level of individuals’ satisfaction with life. The validity and reliability studies were conducted by Ayten (2012) by adapting it to Turkish culture. There are five 7-point Likert-type items on the scale, and high scores indicate high levels of satisfaction with life. In the adaptation study of the scale, the reliability was assessed using the Cronbach’s alpha coefficient, which was found to be 0.85 (Ayten, 2012). A review of studies conducted with the measurement tool indicates that the scale has demonstrated high reliability in other research as well. For instance, in a study by Turan (2018), the Cronbach’s alpha coefficient was reported as 0.82, while in a study by Ayten and Karagöz (2021), it was found to be 0.79. In the present study, the Cronbach’s alpha coefficient was calculated as 0.89, indicating a high level of reliability.

#### 2.3.4 The perception of God scale

The Perception of God Scale was developed by Güler (2007). The validity and reliability studies of the 22-item, five-point Likert-type scale have been conducted. Higher scores reflect a positive perception of God, while lower scores indicate a negative perception. In the development study of the scale, the Cronbach’s alpha value calculated for reliability was found to be 0.83 (Güler, 2007). When examining studies conducted with the measurement tool, it was determined that the scale also produced highly reliable results in other studies. For example, Kiraç (2021) calculated the Cronbach’s alpha value as 0.93 in his study, and Yazıcı Çelebi and Kaya (2023) determined the Cronbach’s alpha value to be 0.92 in their study. The Cronbach’s alpha value for the data obtained in this study was calculated as 0.82, indicating that the results are highly reliable.

### 2.4 Data collection process

The researchers contacted individuals who were registered with official institutions with their contact and residence information and lived alone, using phone calls, social media, or email. Information about the study was provided, and a detailed survey form was shared with the individuals online. The survey included sections informing participants about their rights, including their right to withdraw from the study at any time. The survey was administered between January 2021 and March 2021, and participants took approximately 20 min to complete it. The inclusion criteria for participation in the study were:

- Being an individual aged 40 or older who lives alone at home,
- Being literate and able to provide information about loneliness, life satisfaction, and perception of God,
- Having residence or contact information registered with official institutions.

The exclusion criteria were:

- Lack of internet access,
- Having a psychiatric disorder,
- Not willing to participate in the study.

Using the contact information obtained from official institutions, the researchers attempted to reach approximately 450 individuals. As a result of these attempts, individuals without internet access (50 Participant), those who were illiterate (20 Participant), and those who did not want to participate in the study (2 Participant) were excluded.

### 2.5 Data analysis

Prior to conducting statistical analyses, several preparatory steps were taken. Data integrity and extreme value analyses revealed no missing values in the dataset. For extreme value detection, variable scores were converted to standardized z-scores, and all values were within the acceptable range of −3 to +3 (Tabachnick and Fidell, 2014). Next, skewness and kurtosis values were examined to assess normality. As shown in Table 1, all variables met the normality assumption, with values falling within the range of +2 to −2, consistent with criteria from the literature (George and Mallery, 2019). Descriptive statistics for all variables were subsequently calculated. To test the study’s hypothesis, path analysis was conducted using the IBM AMOS software with the maximum likelihood estimation method. Observed variables were used in the analysis, and predictor and moderator variables were standardized beforehand. Various fit indices were considered to assess how well the model aligned with the data. Accordingly, CFI and NFI values above 0.90, as well as RMSEA and SRMR values below 0.08, were used as benchmarks (Hu and Bentler, 1999; Kline, 2016). An RMSEA value of 0.05 signifies a good fit, values between 0.05 and 0.08 represent an acceptable fit, while those exceeding 0.10 indicate a poor fit (Brown, 2015).

## 3 Results

According to the results of the correlation analysis, there was a significant negative correlation between loneliness and satisfaction with life (*r* = −0.46, *p* < 0.001, 95% CI [−0.53, −0.37]) and the perception of God (*r* = −0.39, *p* < 0.001, 95% CI [−0.49, −0.29]) and a significant positive correlation between satisfaction with life and the perception of God (*r* = 0.40, *p* < 0.001, 95% CI [0.32, 0.49]). The descriptive and correlation values of the variables used in the study are given in Table 2.

**Table 2:** Descriptive statistics and correlations between variables.

Variable	1	2	3
1. Satisfaction with Life	1		
2. Loneliness	−0.46***	1	
3. God Perception	0.40***	−0.39***	1
Female mean	20.37	36.99	87.91
Female SD	7.09	9.48	14.53
Skewness	−0.05	0.80	−0.79
Kurtosis	−0.42	0.94	0.45
Male mean	20.18	38.19	87.76
Male SD	6.40	9.81	14.46
Skewness	−0.10	0.51	−1.08
Kurtosis	−0.23	−0.40	1.18
Total mean	20.28	37.57	87.84
Total SD	6.75	9.65	14.48
Min.	5.00	20.00	36.00
Max.	35.00	77.00	110.00
Skewness	−0.07	0.65	−0.92
Kurtosis	−0.33	0.21	0.78

^***^p < 0.001.

The results of the path analysis conducted with the observed variables regarding the regulatory role of the perception of God in the relationship between loneliness and satisfaction with life are given in Table 3.

**Table 3:** The results of the path analysis showing the regulatory effects.

Parameter	*Β*	*SE*	*t*	*p*	*R^2^*
Loneliness (*X*)	−0.38^***^	0.33	−7.75	<0.001	
God perception (*W*)	0.28^***^	0.32	5.81	<0.001	0.28
X.W	−0.10^*^	0.27	−2.27	0.023	

***p < 0.001, *p < 0.05.

**Figure 1:**
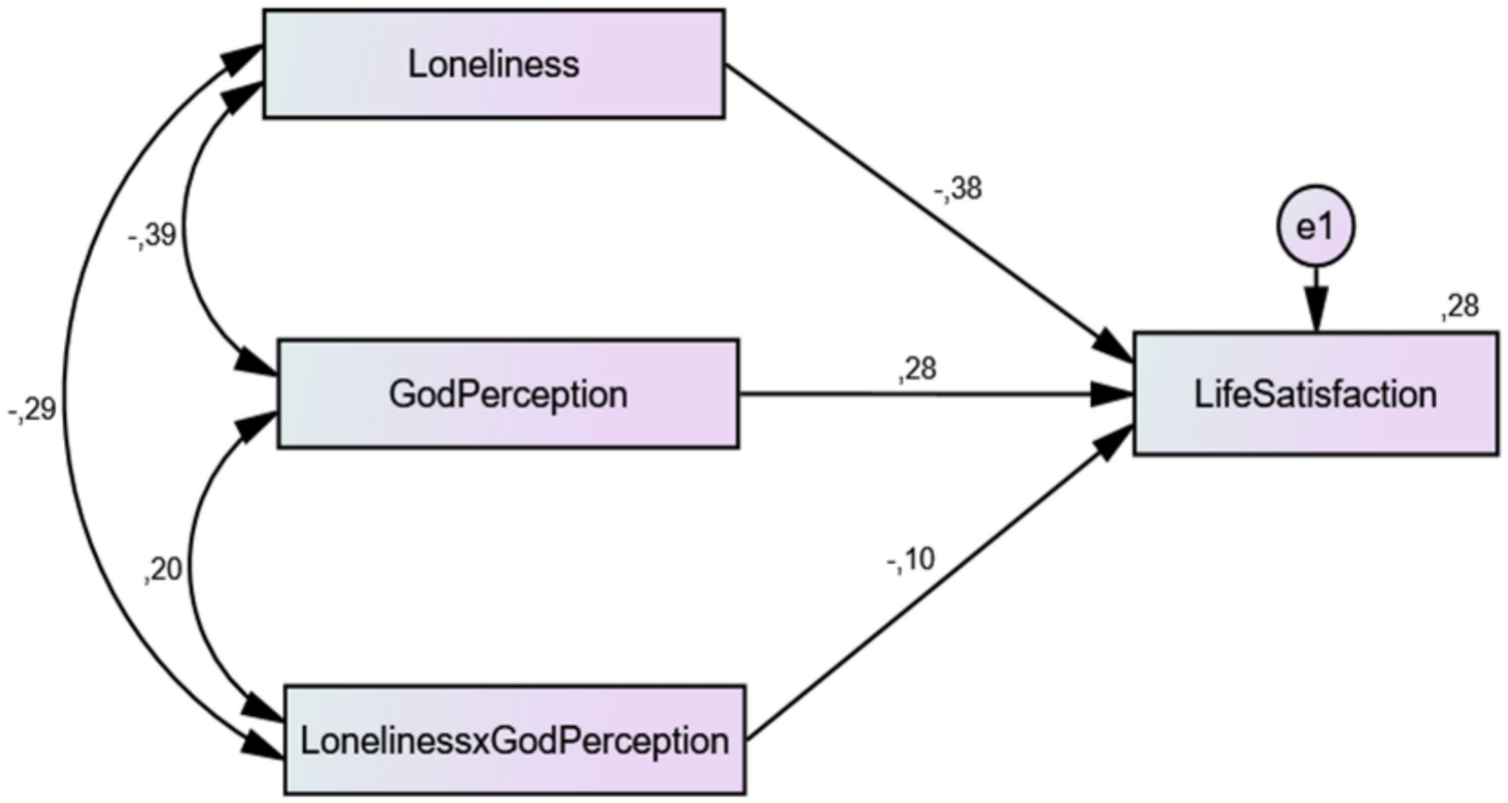
The regulatory role of the perception of God between loneliness and satisfaction with life.

A path analysis was conducted to test the regulatory role of the perception of God regarding the effect of loneliness on individuals’ satisfaction with life. When examining the fit of the path analysis, it was confirmed that the model demonstrated an acceptable fit to the data (NFI = 1.000; CFI = 1.000; RMSEA = 0.088; SRMR = 0.000). When we examined the findings obtained within the scope of the research, we found that the variables of loneliness and the perception of God and the interaction between loneliness and perception of God explained 28% (*R^2^* = 0.28) of the variation in individuals’ satisfaction with life. We determined that the perception of God had a significant positive effect on satisfaction with life (*β* = 0.28, *p* < 0.001) and that loneliness had a significant negative effect on it (*β* = −0.38, *p* < 0.001). The interactional effect (regulatory effect) of loneliness and perception of God variables on satisfaction with life was found to be significant (*β* = −0.10, *p* = 0.023). The results of the path analysis conducted with the observed variables regarding the regulatory role of the perception of God in the relationship between loneliness and satisfaction with life are given in Figure 1.

**Figure 2:**
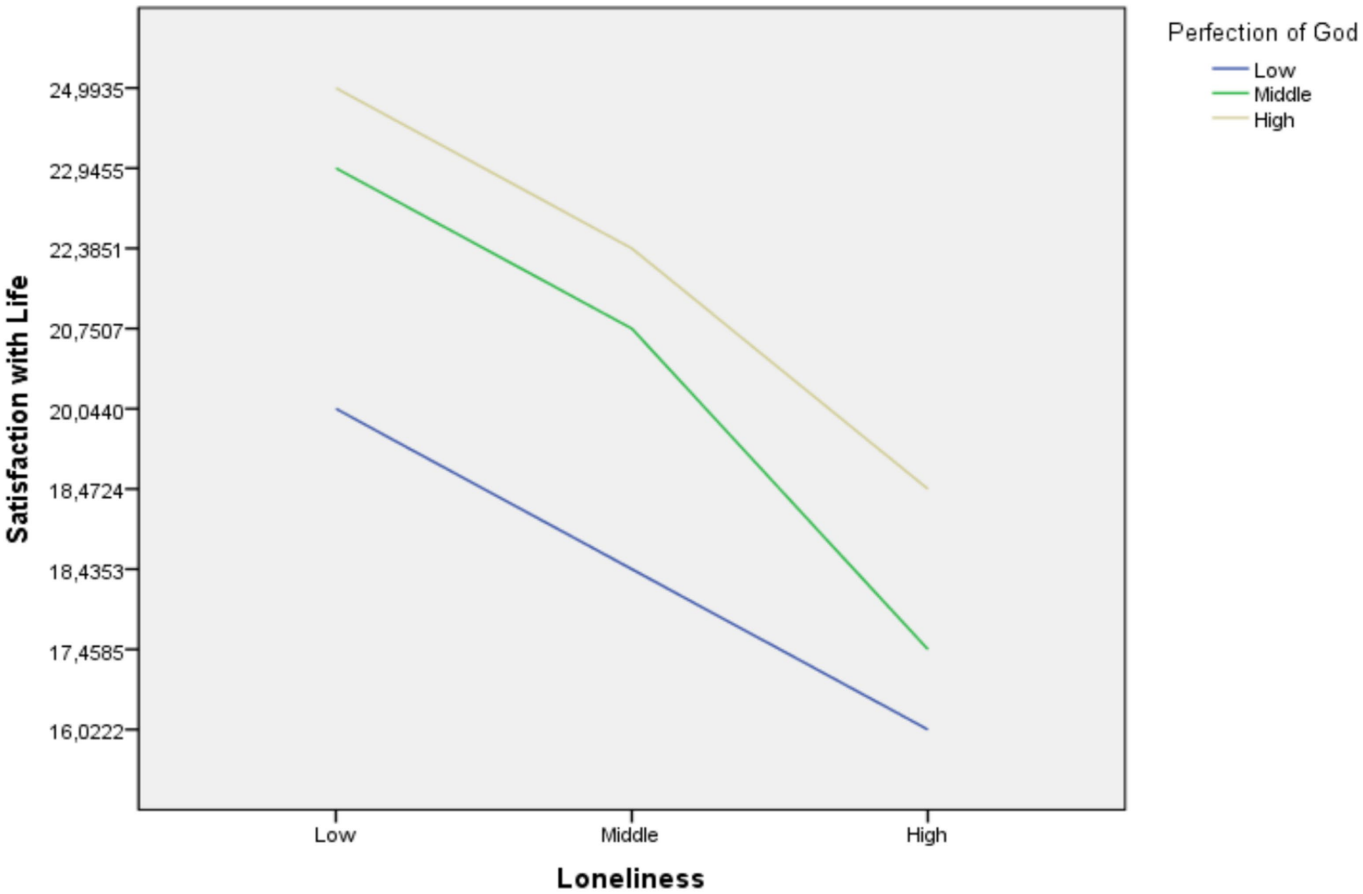
Graphical representation of the regulatory effect of the perception of God.

Since the interactional effect was found significant as a result of the regulatory analysis conducted, a slope analysis was performed. The regulatory effects found as a result of the slope analysis are shown graphically in Figure 2.

When we examined the details of the regulatory effect, we found that the effect of loneliness on satisfaction with life decreased when the perception of God was high (*B* = −3.18, *p* < 0.001). Similarly, the effect of loneliness on satisfaction with life decreased in cases where the perception of God was low (*B* = −1.95, *p* < 0.001). In another result, the effect of loneliness on satisfaction with life decreased in cases where the perception of God was middle (*B* = −2.65, *p* < 0.001). According to the findings, when the perception of God was high, the effect of loneliness on satisfaction with life decreased further. This means that the relationship between loneliness and satisfaction with life is regulated by the perception of God.

## 4 Discussion

In this study, first, the findings regarding the differentiation of loneliness, satisfaction with life, and God perception based on gender were obtained, followed by the relationships between these variables and the moderating findings. These findings were then discussed in the discussion section. The findings obtained from the study indicate that perceptions of God do not differ based on gender. In the literature, different results have been observed in this regard. Along with studies that reached similar conclusions (Atan and Kula, 2024; Erdoğan, 2015), some studies show significant differences between women and men (Baynal and Uysal, 2023; Seyhan, 2014; Yıldız and Ünal, 2017). Another demographic finding of the study indicates that life satisfaction does not differ by gender in the older age group. There are different findings in the literature on this issue. Consistent with this research, some studies have found no gender-based differences (Altay and Avcı, 2009; Eshkoor et al., 2015; Tian and Chen, 2022). However, other studies suggest that women have higher life satisfaction (Della Giusta et al., 2011; Joshanloo and Jovanović, 2020; Kutubaeva, 2019), while some indicate that men report higher life satisfaction (Matud et al., 2014). The final analysis conducted in the study with demographic variables also indicates that loneliness levels do not differ by gender. Supporting studies (Gul et al., 2018; Maes et al., 2019) suggest that there is no gender-based difference in loneliness levels, while some studies show that women experience higher levels of loneliness (Doğan and Başer, 2019; Perlman, 2004). On the other hand, Lorber et al. (2023) report that loneliness levels are higher in men. The variation in results may stem from differences in the age groups of the samples, differences in gender perceptions, and interactions between gender and other variables. In this study, the focus on adult and elderly individuals may have led to gender-based differences being less prominent.

The results obtained from the study showed that there was a significant negative correlation between loneliness and satisfaction with life and that loneliness significantly predicted satisfaction with life. Accordingly, the first hypothesis of the study was confirmed. In the literature, many studies have shown a negative relationship between loneliness and satisfaction with life (Bozorgpour and Salimi, 2012; Chow, 2005; Doman and Le Roux, 2012; Ekşi et al., 2020; Hu et al., 2016; Huebner et al., 2010; Kearns et al., 2015; Lee and Ishii-Kuntz, 1987; Lim and Kua, 2011; Yukay-Yüksel et al., 2020). Altay and Çalmaz (2023), in their study conducted with older adults during the COVID-19 pandemic, found that isolation negatively impacted satisfaction with life. Social isolation disrupted or significantly limited support mechanisms that are important for individuals, leading to feelings of loneliness. This situation posed greater risks, particularly for older adults (Şahan et al., 2023). Research has shown that satisfaction with life is influenced by factors such as loneliness and social support (Cowlishaw et al., 2013; Ozmen et al., 2018; Ranta et al., 2013; Røysamb et al., 2003); higher satisfaction with life is associated with better social relationships (Koivumaa-Honkanen et al., 2001); and satisfaction with life is affected by interactions such as social support and social bonds (Segrin, 2006). Additionally, lonely individuals tend to have lower satisfaction with life (Goodwin et al., 2001; Swami et al., 2007). A study conducted by Bhutani and Greenwald (2021) revealed that loneliness had several negative consequences for older adults in the context of COVID-19. One of these consequences is the decline in satisfaction with life. Numerous studies have demonstrated that loneliness is a predictor of satisfaction with life, and these findings are consistent with the results of this study (Çivitci and Çivitci, 2009; Lorber et al., 2023; Vanhalst et al., 2013). Loneliness is associated with negative aspects of mental health (Eisses et al., 2004; Nangle et al., 2003; Stravynski and Boyer, 2001). When these findings were evaluated in terms of cognitive theory, individuals who were leading a normal life were put into quarantine in addition to the negative situation experienced and continued their lives alone for a certain period. The negative cognitions that individuals attributed to loneliness in this process might have caused them to perceive their satisfaction with life more negatively as a result. In other words, negative evaluations about situations that increased loneliness, such as the decrease in face-to-face interactions with others compared to the pre-pandemic period, problems with participating in social areas, and separation of individuals from each other due to the fear of infection, may have caused individuals to get less satisfaction with life. From this point of view, it is an expected result that loneliness is negatively related to satisfaction with life.

The results obtained from the study showed that there was a significant positive correlation between the perception of God and satisfaction with life and that the perception of God significantly predicted satisfaction with life. In this context, the second hypothesis examined in the study was confirmed. There are similar research findings showing a significant correlation between the perception of God and satisfaction with life (Hosseinsabet and Rady, 2015; Zahl and Gibson, 2012). From a broader perspective, we see that there are studies in the literature revealing that the perception of God is correlated with positive psychological elements. For example, Dilmaç and Çiftçi (2019) concluded that a positive perception of God was associated with psychological resilience. Maton (1989) and Schaefer and Gorsuch (1991) found that there was a positive relationship between positive perception of God and psychological health and a negative relationship with anxiety. Studies investigating the relationship between perception of God and mental health (Bradshaw et al., 2010), psychological well-being (Seyhan, 2014), and satisfaction with life (Zahl and Gibson, 2012) have indicated that positive perception of God is positively associated with positive psychological characteristics. In addition, some studies have shown that there is a positive relationship between negative perception of God and anxiety and trauma (Justice and Lambert, 1986; Kane et al., 1993). When we evaluated this finding in terms of cognitive theory, we concluded that the strength found in the presence of God during the lockdown process and feeling that God was always by their side by individuals who considered God as the source of love and a forgiving and protective power would also positively affect their evaluations of their lives in general. Indeed, researchers have reported that having strong beliefs about the existence of God has a positive effect on the individual’s well-being by reducing cognitive dissonance (Villani et al., 2019). We can state that the positive perception of God by individuals who are in the process of social isolation and whose life processes have changed contributes to them feeling less lonely in this process and, as a result, getting more satisfaction from life. Enea et al. (2021) found that during the COVID-19 period, people’s levels of loneliness and their engagement with matters related to God increased.

The results obtained from the study showed that the interaction between loneliness and the perception of God significantly predicted satisfaction with life. In this context, the third hypothesis examined in the study was confirmed. The results of the research have shown that there is an inverse relationship between the perception of God and loneliness. In other words, individuals with a positive perception of God have lower levels of loneliness. The belief in God in Islam is that God is always everywhere. We can say that people who have strong faith can feel that God is with them and that they think they are in constant communication with God through prayers or worship, which will reduce the feeling of loneliness. In a similar study, the relationships between loneliness, perception of God, and dysfunctional attitudes were examined, and the researchers found that there was a negative relationship between perception of God and loneliness (Mohammadpour et al., 1985). Similarly, Kirkpatrick et al. (1999) and Le Roux (1998) concluded that spirituality reduced the level of loneliness. When the result of the research regarding the moderator finding is considered in terms of cognitive theory, we can say that the individual’s positive perception of God and the idea that God is a protective power that is always with him/her will affect his/her evaluations of loneliness and reduce the feeling of loneliness. Thus, we can state that individuals’ satisfaction with life will be affected less negatively. This situation seems to be compatible with the understanding of the cognitive approach that connects the basis of emotions to thoughts. The positive perception of God can be associated with alleviating the negative effects of loneliness on satisfaction with life and the tendency of the individual’s evaluations of his/her own life to be positive. Muhammad et al. (2023), in their study conducted with older adults in India, found that elderly individuals who felt lonely reported lower satisfaction with life. They also concluded that the negative impact of loneliness on satisfaction with life was mitigated by religiosity, spirituality, and religious participation. Similarly, another study conducted with a Christian sample demonstrated that closeness to God predicts satisfaction with life (Culver, 2021). These findings are consistent with the results of this study and also highlight the importance of representations of the God figure-how individuals conceptualize God in their minds-regardless of the religion they belong to. Based on all these research findings, we can say that positive perception of God is associated with psychological health and positive emotional status and plays a protective role. Research findings have shown that the perception of God has a regulatory role between loneliness and satisfaction with life. In the literature, no research findings on this subject were found. In this context, we think that the findings of the current research will contribute to the field. Loneliness is negatively correlated with satisfaction with life. As the level of loneliness increases, satisfaction with life decreases, and the perception of God affects this relationship. We can say that individuals who have a positive perception of God, that is, who perceive God as forgiving and reassuring, who think they are in communication with God through prayers, worship, or similar ways, and who feel that God is always by their side as a supporting power, have lower levels of loneliness, and that this positively affects their satisfaction with life. In light of these findings, it can be suggested that a faith-sensitive therapeutic approach may be effective when addressing issues such as loneliness or satisfaction with life during the therapy process. Experimental studies could be recommended to test the effectiveness of incorporating the client’s belief system and spiritual practices (such as prayer, worship, meditation, etc.) into therapy. Additionally, we think that studies conducted with larger sample groups and different variables will be beneficial for ensuring the generalizability of the results. The Turkish society, which forms the sample of this study, is a collectivist society. Therefore, variables such as social isolation and loneliness need to be addressed within this context. The reflection of these variables may be different in individualistic societies. For this reason, we believe that cross-cultural comparative studies will contribute to the literature. Policies could be developed to protect middle-aged and older adults from the negative effects of loneliness, and support programs could be created to encourage socialization and integration into life. Structured group counseling programs for elderly individuals could be implemented in the field of social services.

In this study, there are a few additional limitations I would like to express. First, the sample consisted of 378 participants residing in the provinces of Gümüşhane, Bayburt, and Erzurum, predominantly Muslims. Future studies could include a larger and more diverse sample from various regions and religious backgrounds to provide broader insights into the moderating role of God perception in the relationship between loneliness and satisfaction with life. Another limitation is that the data were collected from elderly participants who were literate and had access to the internet, which may not represent the wider elderly population. We can say that literacy may cause differences compared to individuals who are not literate in many aspects, especially in terms of following the developments in the COVID-19 process. At this point, the results of the current study can be generalized by examining the relationships between loneliness, perception of God, and satisfaction with life in individuals who are not literate and do not have internet access.

## Data Availability

The raw data supporting the conclusions of this article will be made available by the authors, without undue reservation.
